# Priority skills for equity-focused, evidence-based cancer control in community-based organizations: A group concept mapping analysis with academics and practitioners

**DOI:** 10.1017/cts.2023.586

**Published:** 2023-07-10

**Authors:** Shoba Ramanadhan, Maggie Weese, Scott R. Rosas, Jennifer L. Cruz, Cindy Chwa, Madison K. Rivard, Shinelle Kirk, Albert Whitaker, Judi Kirk, Karen Peterson, Arthur Eisenkraft

**Affiliations:** 1 Department of Social and Behavioral Sciences, Harvard TH Chan School of Public Health, Boston, MA, USA; 2 Concept Systems, Inc., Ithaca, NY, USA; 3 SUNY Upstate Medical University, Syracuse, NY, USA; 4 Conservation Law Foundation, Boston, MA, USA; 5 American Heart Association, Waltham, MA, USA; 6 St. Mark Congregational Church, Boston, MA, USA; 7 Boys and Girls Club of Worcester, Worcester, MA, USA; 8 Tufts Medicine, Burlington, MA, USA; 9 University of Massachusetts Boston, Boston, MA, USA

**Keywords:** Evidence-based interventions, community, community-based organizations, skills, capacity, workforce

## Abstract

**Introduction:**

Community-based organizations (CBOs) are important equity-promoting delivery channels for evidence-based interventions (EBIs). However, CBO practitioners often cannot access needed support to build EBI skills. Additionally, the capacity-building literature is hindered by inconsistent definitions, limited use of validated measures, and an emphasis on the perspectives of EBI developers versus implementers. To address these gaps, we explored commonalities and differences between CBO practitioners and academics in conceptualizing and prioritizing core EBI skills.

**Methods:**

We utilized Group Concept Mapping, a mixed-methods approach connecting qualitative data (e.g., regarding the range of critical EBI skills) and quantitative data (e.g., sorting and ranking data regarding unique skills) to create conceptual maps integrating perspectives from diverse participants. A total of 34 practitioners and 30 academics working with cancer inequities participated in the study.

**Results:**

Participants nominated 581 core skills for EBI use, and our team (including practitioners and academics) identified 98 unique skills from this list. Participants sorted them into conceptual groups, yielding five clusters: (1) using data and evaluation, (2) selecting and adapting EBIs, (3) connecting with community members, (4) building diverse and equitable partnerships, and (5) managing EBI implementation. The ordering of importance and presence of skill clusters were similar across groups. Overall, importance was rated higher than presence, suggesting capacity gaps.

**Conclusions:**

There are helpful commonalities between practitioners’ and academics’ views of core EBI skills in CBOs and apparent capacity gaps. However, underlying patterns suggest that differences between the groups’ perceptions warrant further exploration.

## Introduction

Many implementation strategies (the tactics used to support the integration of research evidence into routine practice) include efforts to train implementers [1]. However, in community-based organizations (CBOs), practitioners often do not receive sufficient training on the use of evidence-based interventions (EBIs), which constrains impact [2]. This is particularly important given that CBOs can deliver EBIs to address health inequities by leveraging their reach and trust among groups ineffectively served by typical public health and healthcare channels [2,3]. Interventions to build capacity (skills, knowledge, motivation, resources, self-efficacy, and awareness) to find and use EBIs offer a significant potential solution [4]. However, a series of gaps hinder progress in developing and testing effective capacity-building implementation strategies.

First, the bulk of the capacity-building literature focuses on skills held by staff of agencies with vastly different organizational contexts, resources, and professional development opportunities compared to CBOs (such as health departments) [5–7]. Second, within the literature focused on CBOs, a recent scoping review highlights inconsistent definitions and limited use of validated capacity-building measures, which restricts progress [8]. Finally, and most importantly, the literature and national programs emphasize the following skills: assessing context/needs, engaging partners, and selecting, adapting, integrating, evaluating, and sustaining EBIs [4,9,10]. These skills reflect many of the core principles of evidence-based public health [2]. Yet, our recent qualitative research with academics and CBO practitioners suggests that while this list resonated with both groups, there are important nuances and potential additions to the list. Participants added cultural humility, communication, and systems change as skills necessary for EBI use when addressing cancer equity. At the same time, the fundamental orientations to skill conceptualizations highlighted a strong disconnect. CBO practitioners grounded their discussions of skills in community needs and long-term health promotion goals. In contrast, academics tended to focus on the EBI and other system attributes related to the technology being transferred [11]. Over the last decade, our team and colleagues have repeatedly found that CBO practitioners and academics often understand research evidence, EBIs, and opportunities to build capacity for EBI use in meaningfully different ways [12–14]. These findings suggest further exploration of the core set of EBI skills is warranted.

We furthered our exploration of these issues using US cervical cancer inequities as an exemplar. This was a suitable choice for two reasons. First, despite the rich evidence base related to prevention (e.g., vaccination and screening), in 2018 there were about 12,000 new cases of cervical cancer, with disproportionate disease burden among racial and ethnic minorities, populations of low socioeconomic status, and those living in rural areas [15–17]. Second, CBOs can play an essential role in addressing community demand and access (key levers highlighted by the Community Preventive Services Task Force) [18], but the gap in capacity is an important limiting factor.

We used group concept mapping to identify a parsimonious, prioritized list of skills for potential capacity-building interventions that draw on practitioner and academic expertise. Group concept mapping is a mixed-method approach that supports the development of a shared conceptualization among diverse participants [19]. This method is becoming increasingly common in implementation science, e.g., supporting the development of the Expert Recommendations for Implementing Change typology of implementation strategies and identifying alignment between implementation science and user-centered design [20,21]. In addition to understanding the conceptualization of core skills for EBI use in CBOs among practitioners and academics, we also sought to explore the extent to which these skills were considered important and available in CBO settings.

## Materials and Methods

### Group Concept Mapping Overview

**Figure 1.**
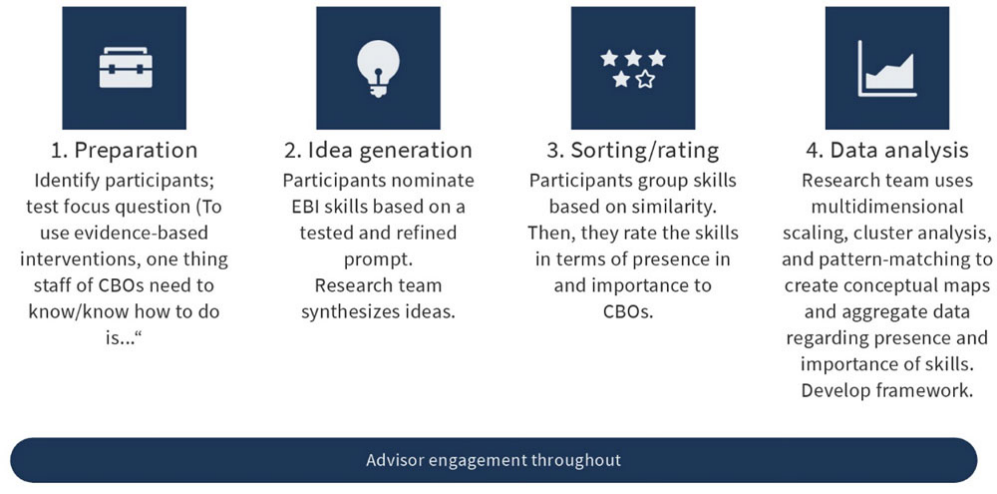
The flow of activities in data collection and analysis conducted by the research team, with guidance from community advisors, adapted from Rosas, 2017 [23]. CBO = community-based organization; EBI = evidence-based intervention.

Given our goal of developing a shared conceptual framework, we chose to use group concept mapping, which integrates qualitative group processes with multivariate statistical analyses [19,22]. The approach allows groups to articulate and delineate concepts and their interrelationships and is frequently used to explore the complexity of social phenomena [23]. Fig. 1 summarizes the flow of activities using the groupwisdom^TM^ web-based platform [24].

The point map uses two-dimensional space to display the conceptual relationship between statements, with proximity between statements reflecting how often participants sorted the statements together. This map serves as the base for the cluster map, which utilizes Ward’s algorithm to partition the map into non-overlapping clusters or groups of items that presumably represent meaningful concepts based on the proximal location of statements. To gather input on the perceived values of the ideas on the map, participants rated each statement based on its presence in CBOs and its importance for supporting staff of CBOs to use EBIs. The software also represents the data using pattern match graphs (ladder graphs) and bivariate scatter plots that display participants’ ratings of the ideas for comparative purposes [25].

### Phase 1 – Preparation

As reported in detail elsewhere, we conducted semi-structured interviews with 8 CBO staff members, a funder, and 11 academics to explore each participant’s perspective on the skills CBO staff need to implement cervical cancer prevention EBIs to improve outcomes for marginalized populations. At a high level, we focused on recruiting staff and researchers with expertise in their field whose work addressed cancer inequities among marginalized populations. We recruited a diverse sample in terms of US region and populations served. The one-hour semi-structured interview emphasized the range of skills required in CBOs to use EBIs. For this analysis, we pulled lists of suggested skills from transcripts to complement the Phase 2 idea generation activities.

### Phase 2 – Web-based Idea Generation

We utilized the online platform groupwisdom^TM^ to collect ideas from participants regarding the skills necessary to implement evidence-based interventions. Group concept mapping data were collected from June to August 2021.

Participants: We used a purposive sampling approach, which is typical for group concept mapping studies and allows for targeted recruitment to gather a diversity of perspectives [19]. We recruited from networks held by the project team, advisory panel, and community partners. We also recruited participants via state cancer coalitions, the National Cancer Institute Cancer Consortium for Implementation Science, the University of Massachusetts Dana-Farber/Harvard Cancer Center Partnership, and nominations from participants. Recruitment focused on participants whose work addressed cervical cancer among marginalized populations. Participant eligibility requirements for CBO staff members included having worked in public health for at least five years and implementing at least one evidence-based cervical cancer prevention program. Participant eligibility requirements for academic researchers included at least five years of experience designing, implementing, or evaluating evidence-based cancer prevention programs, including at least one focused on cervical cancer.

Activities: As participants began the activity, the study team prompted them to consider their equity-focused work, particularly cervical cancer-related work, while offering responses. They then responded to a refined focus prompt: “To use evidence-based interventions, one thing staff of community-based organizations need to know/know how to do is …” The prompt was tested and refined through the Phase 1 key informant interviews to ensure that responses fit within our research goals. Participants submitted their ideas anonymously and could see an up-to-date list of all participant ideas, including their own. The activity was open for 31 days, allowing participants to submit ideas, leave, and return to the idea generation activity as desired.

Analysis: In total, participants provided over 581 statements. Following the multi-step idea synthesis process outlined in Kane and Rosas [25], the project team reduced the complete set of statements to a final list of 98 using the following criteria: (a) relevance to the focus question, (b) redundancy or duplication, and (c) clarity of meaning. The project advisory group offered edits to the preliminary and final list of statements to support application of the criteria and utilize language that would resonate with a broad range of participants.

### Phase 3 – Sorting and Rating

The focus of Phase 3 was to capture participants’ groupings of the reduced list of skills and their perceptions of the extent to which these skills were important for and available in CBO settings.

Participants: Same as Phase 2.

Activities: Participants reacted to the list of 98 statements that represented the pool of ideas generated earlier. The statement sorting and rating activity was open for 31 days. For the sorting activity, participants sorted the list of 98 statements into groups “in a way that makes sense to [them].” Given that it is an unstructured sort, there was no pre-determined number of groups or piles that participants were expected to make. The only restrictions in the sorting task were that there could not be: (a) as many piles as there were statements, i.e., each pile having only one item in it; (b) one pile consisting of all statements; or (c) a “miscellaneous” pile that included statements that could not otherwise be grouped. Participants also named each pile with a title that captured the general theme of the statements contained.

For the rating activity, participants rated each of the 98 statements on a five-point Likert-like scale for two variables: importance and presence. The importance variable supports prioritization of skills. The presence variable supports identifying what is available in CBO settings. Together, they point to gaps to be addressed in future interventions [25]. For importance, participants rated each statement in terms of how important the skill is for supporting staff of community-based organizations to use evidence-based interventions, where 1 is “not important at all” and 5 is “extremely important.” For presence, participants were asked to rate each skill in terms of how commonly this skill is found in community-based organizations they are familiar with, where 1 is “I never see evidence of this skill” and 5 is “I always see evidence of this skill.” During this phase, participants were also asked a series of background questions, including which groups from the NIH list of priority populations were commonly included in their research or practice (if any), how many years of research and/or practice experience they had, and demographic questions.

Analysis (sorting): Utilizing groupwisdom^TM^, we created a similarity matrix for each participant in the form of a 98 × 98 matrix (one column and one row for each statement). For each cell, a “1” was placed at the intersection of two items if the participant grouped them and a “0” if the participant did not group the items together. The individual participant matrices were aggregated into a combined matrix, which was analyzed using non-metric multidimensional scaling analysis with a two-dimensional solution [26]. This analysis supported the creation of a two-dimensional map that used a single point to represent a statement and coordinates to represent the placement of the statement in relation to others. Thus, statements that appear closer together were more likely to have been sorted together than statements that are far apart on the map.

The x, y coordinates for each point were then used as the input for the hierarchical cluster analysis utilizing Ward’s algorithm [27] as the basis for defining a cluster. Using the multidimensional scaling configuration as input to the cluster analysis forces the cluster analysis to partition the scaling configuration into non-overlapping clusters in two-dimensional space. Although there is no defined optimal number of clusters, evaluation of a range of cluster configurations to determine the solution that best represents the data and participants’ organization of items is warranted. Through an iterative review process suggested by Kane and Trochim [22], we started with a solution involving 10 clusters. We merged clusters until we found a cluster solution that was meaningful, efficient, and avoided merging dissimilar concepts. We also referred to the interviews from Phase 1 to understand how practitioners and academics discussed needed skills for EBI use.

Analysis (rating): In addition to the cluster map solution, we created graphical representations of the statement and cluster rating data through ‘go-zone’ maps and ‘ladder’ graphs. The go-zone map is a bivariate x,y graph that plots each statement using its average importance rating (*x*-axis) and average presence rating (*y*-axis) and is then divided into quadrants. The ladder graph also compares average ratings; however, unlike the go-zone map, it compares the average rating of each cluster instead of each statement. Analysis in groupwisdom^TM^ displays the ladder graph using two side-by-side vertical axes, comparing clusters across participant groups.

### Phase 4 – Analyzing and Interpreting

Analysis activities are detailed above with the sorting and rating activities. The research team collaboratively interpreted data with three community advisors with rich expertise in implementing EBIs in community settings to address health equity (AW, KP, JK). We solicited feedback from these advisors to define the project’s scope, evaluate the pared-down list of statements after the idea generation phase, review and interpret the cluster solution, and determine how best to share results with participants and other stakeholders. They also participated in the development of this manuscript and are co-authors. One advisor joined later than the others and contributed to the final two feedback activities. We also shared preliminary results with all participants in a brief that included an invitation to comment and share interpretations. We received feedback from 7 of the 64 participants identifying points of interest and ideas for the next steps in the research.

### Ethics Approvals and Study Incentives

The Harvard University IRB reviewed all study procedures and materials, and the study was deemed exempt from review. At each stage, participants gave their consent for participation. All participants were offered gift cards of $50 for interviews, $75 for idea generation, and $100 for the sorting/rating activity.

## Results

The group concept mapping activities included 64 participants, described in Table 1. On average, participants had 17 years of research and/or practice experience. As highlighted below, participants conducted research and practice-based work with a wide range of communities experiencing health inequities. A total of 64 of the 148 individuals we contacted for our purposive sample agreed to participate. The 43% response rate is consistent with group concept mapping exercises [28].

**Table 1.** Characteristics of participants in the group concept mapping activities (n = 64)

	Academics (*n* = 30)	CBO Practitioners (*n* = 34)	Total (*n* = 64)
Region			
South	16	11	27
Northeast	9	14	23
West	3	2	5
Midwest	2	7	9
Populations of focus*			
Low-income	24	24	48
Black or African American	21	22	43
Latinx/Hispanic	19	21	40
Rural	17	15	32
Asian	8	11	19
LGBTQ+	7	15	22
American Indian or Alaska Native	5	8	13
Pacific Islander	2	4	6
Participant race*			
White	24	17	41
Black or African American	1	10	11
Asian	4	3	7
American Indian or Alaska Native	0	2	2
Other race	1	2	3
Prefer not to say	0	2	2
Ethnicity			
Hispanic / Latinx	3	3	6
Years of research and/or practice experience (Mean, standard deviation)	20.83 (10.14)	13.78 (8.31)	17.14 (9.81)

* Multiple selections permitted.

### Idea Generation Results

A total of 372 responses were offered through the web-based brainstorming activities and 142 from the interviews. Following the steps outlined by Kane & Rosas [25], the team conducted an idea synthesis process, which included editing the responses for clarity or grammatical errors and splitting responses that contained multiple ideas into statements that contained a single idea. This process yielded 581 statements, which were then coded by keyword. Duplicate items were dropped and then the study team, in conjunction with advisors, worked to finalize the list, focusing on capturing the diversity of ideas offered and ensuring that the language would resonate with practitioners and academics. The final list included 98 items, which fit within the goal of 80-100 statements suggested by Kane and Trochim [22].

### Sorting Results

**Figure 2.**
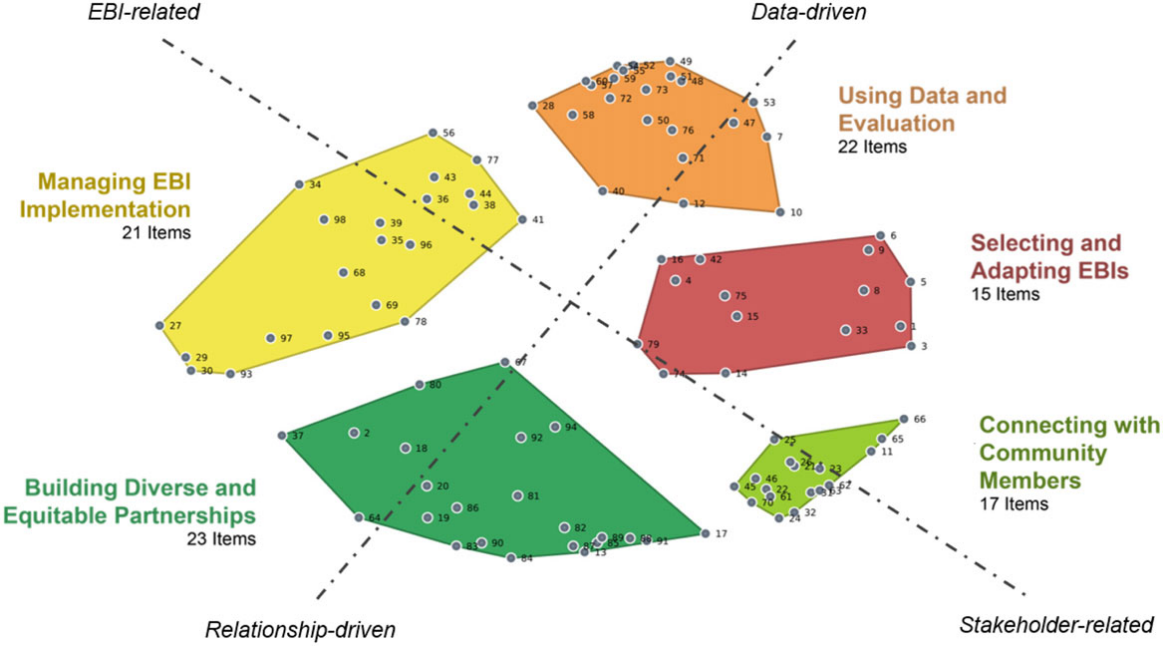
Distribution of 98 statements capturing skills for EBI use across 5 clusters (*n* = 64 participants). EBI = evidence-based intervention; dashed lines delineate potential dimensions of interest.

Participants sorted statements into groups that seemed meaningful to them. After a systematic review of multiple solutions, a five-cluster solution was identified as the most parsimonious depiction of the point arrangement, as shown in Fig. 2. Each gray dot represents a statement and the distance between any pair of dots reflects the extent to which they were or were not grouped together by participants. The multidimensional scaling analysis converged after 14 iterations and yielded a final stress value of 0.23 indicating good fit between the sort data in the similarity matrix and the point map representation [28].

The list of items in each cluster is included in Supplementary File 1 and we offer a brief description and an exemplar here. The “Using data and evaluation” cluster included skills to evaluate adaptations and process and impact outcomes. An exemplar skill was “how to develop and use ongoing monitoring, audit and feedback systems to collect data throughout the implementation.” The “Selecting and adapting EBIs” cluster emphasized the need to choose an appropriate EBI and then adapt it to balance local and organizational needs while attending to the fidelity of program delivery. An exemplar skill was “how to adapt the intervention to their specific population/situation without losing the fidelity of the intervention.” The “Connecting with community members” cluster emphasized skills related to centering the community receiving services and ensuring community voice impacts EBI delivery. An exemplar skill was “how to create bi-directional channels of communication with communities being served to get insight, feedback, and buy-in.” The “Building diverse and equitable partnerships” cluster emphasized skills relating to collaboration and Partnership with a wide range of stakeholders, including those in healthcare, public health, and academic institutions. The cluster also included skills related to relationship development and management. An exemplar skill was “how to demonstrate dependability & trustworthiness to partners.” The “Managing EBI implementation” cluster included a range of skills related to understanding how the EBI works and seeking and utilizing the necessary resources to support it. An exemplar skill was “how to access appropriate technical assistance and implementation guidance as needed.”

In addition to understanding the clusters of items, we also examined the map to understand the broader patterns present in the configuration. Two dimensions were interpreted along separate axes based on the arrangement of points and clusters (see Fig. 2). One potential axis of interest connected “Building diverse and equitable partnerships” and “Using data and evaluation.” Moving from the center outward, items are gradually stronger indicators of the dimension. Thus, items on the relationship-driven side of the axis indicate a gradual transition from focusing on the organization’s actions related to leadership (closer to the center of the map) to external partnership influences (farther from the center). Items on the data-driven side of the axis emphasize a gradual transition from data related to implementation (closer to the center of the map) to outcomes and equity (farther from the center).

Another potential axis of interest connected “Managing EBI implementation” and “Connecting with community members.” This can be described as attending to the requirements of the EBI as developed on one end and the requirements shared by community members and stakeholders on the other. Items on the EBI-related side of the axis suggest a gradual transition from a focus on understanding EBI elements (closer to the center of the map) to administrative skills (farther from the center of the map). Items on the stakeholder-driven side of the axis related to connecting with community members emphasize a gradual transition from centering and relating to community (closer to the center of the map) to communication-related adaptations made to the EBI (farther from the center of the map).

### Importance and Presence Ratings

**Figure 3.**
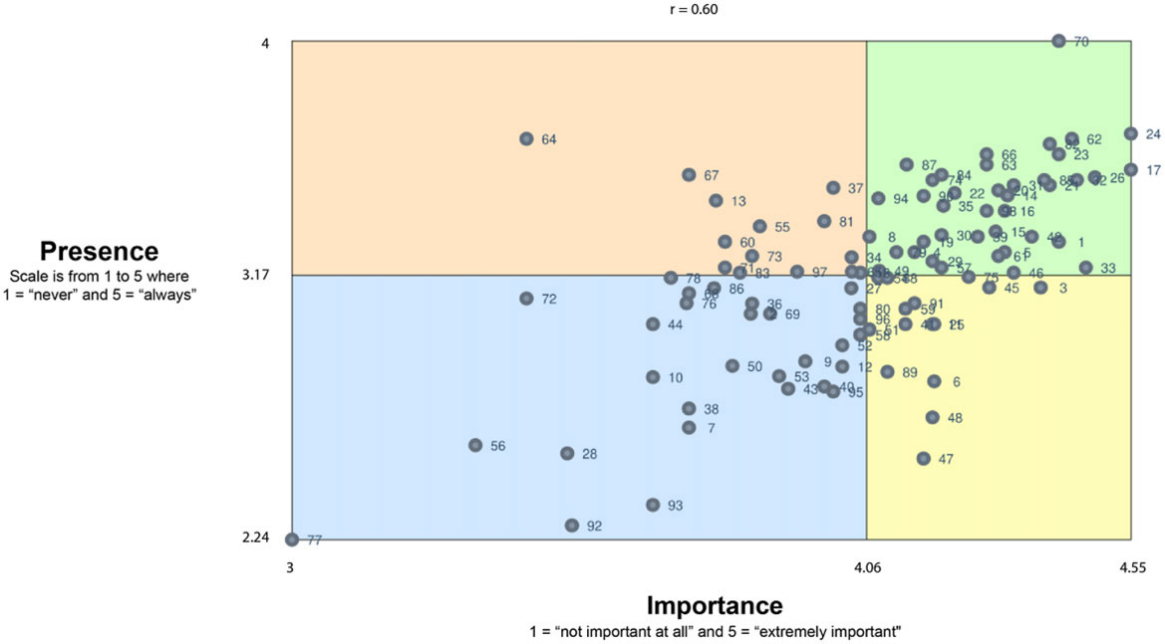
Go-zone map for comparison of importance and presence of 98 statements capturing skills for EBI use (*n* = 64 participants). EBI = Evidence-based intervention.

We utilized a “go-zone” map to compare the importance and presence among all the rated statements. As seen in Fig. 3, skills are presented in quadrants based on a comparison of importance and presence, with the lines demarcating quadrants determined by the mean values of the two ratings. The correlation value (*r* = 0.60) indicates a moderately strong, positive relationship between item-level ratings of importance and presence. Encouragingly, there were 40 skills in the green zone (the quadrant representing above-average importance and presence). The yellow quadrant is of greatest interest from a capacity-building standpoint, which includes skills that participants ranked as higher in importance but lower in presence. Of the 15 statements in this quadrant, five addressed equity considerations, e.g., how to evaluate the implementation process with an equity lens and how to use evaluation to ensure equity in program reach. Three focused on community engagement, emphasizing ongoing engagement and participation of community members in EBI processes. Another three addressed evaluation, two addressed adaptation for local context, one addressed ongoing stakeholder engagement, and one addressed EBI selection. The statements are labeled with numbers in Fig. 3 and can be matched with the list in Supplementary File 1 for further exploration.

We also examined how presence and importance were rated differently by cluster. As seen in Table 2, the average rated importance was higher than the average rated presence, with statistically significant differences for each of the five clusters, even after adjustment of the p-value due to multiple comparisons. In other words, the level of the presence of skills was perceived to be significantly lower than the perceived importance of these skills across all clusters of items.

**Table 2.** Comparison of average rated importance and presence by cluster (n = 64 participants, 98 statements)

Cluster	Importance – average (variance)	Presence – average (variance)	T-value
Selecting and adapting EBIs	4.25 (0.02)	3.24 (0.03)	17.09*
Connecting with community members	4.33 (0.02)	3.43 (0.07)	12.87*
Using data and evaluation	3.91 (0.04)	2.97 (0.06)	14.12*
Building diverse and equitable partnerships	4.04 (0.07)	3.30 (0.09)	8.72*
Managing EBI implementation	3.90 (0.10)	3.00 (0.10)	9.29*

* p-value<0.001; EBI = Evidence-based intervention.

**Figure 4.**
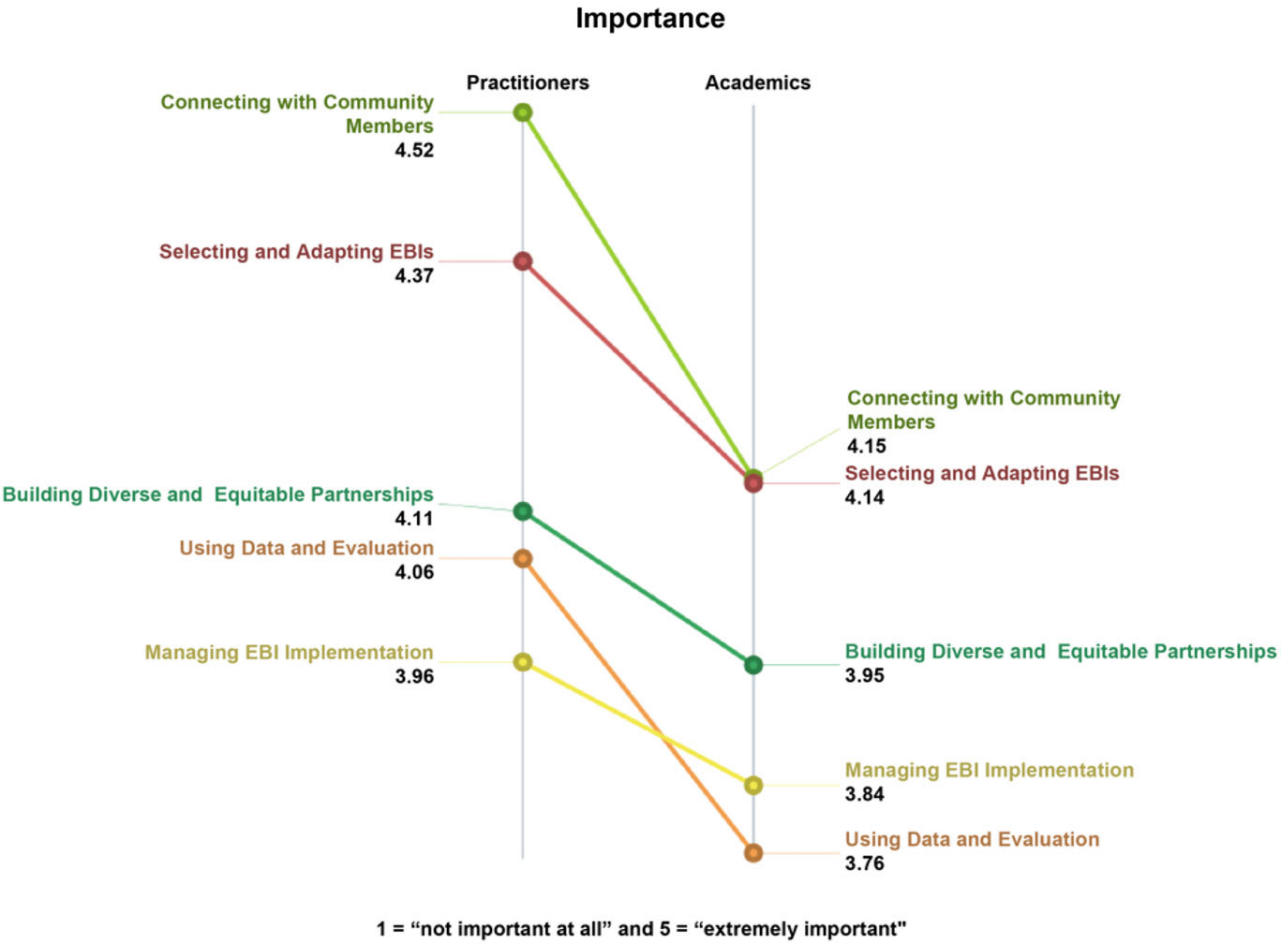
Perceived importance of skills for EBI use in CBOs, by cluster and participant type (*n* = 64 participants, 98 statements). EBI = evidence-based intervention; CBO = community-based organization.

As seen in Fig. 4, although CBO practitioners (overall *M* = 4.18, *SD* = 0.75, Range = 1.35) tended to rate items on the scale of importance higher than academics (overall *M* = 3.95, *SD* = 0.85, Range=1.85*)*, the distribution of average ratings were comparable between the two groups. Thus, we observed consistency between the two groups in the average rating scores across all items. Moreover, the ordering of clusters between the two groups was quite similar, except for the “Using Data and Evaluation” and “Managing EBI Implementation” clusters. The pattern match correlation, which describes the pattern of average cluster ratings across groups, was 0.91, indicating a high magnitude of agreement between groups.

**Figure 5.**
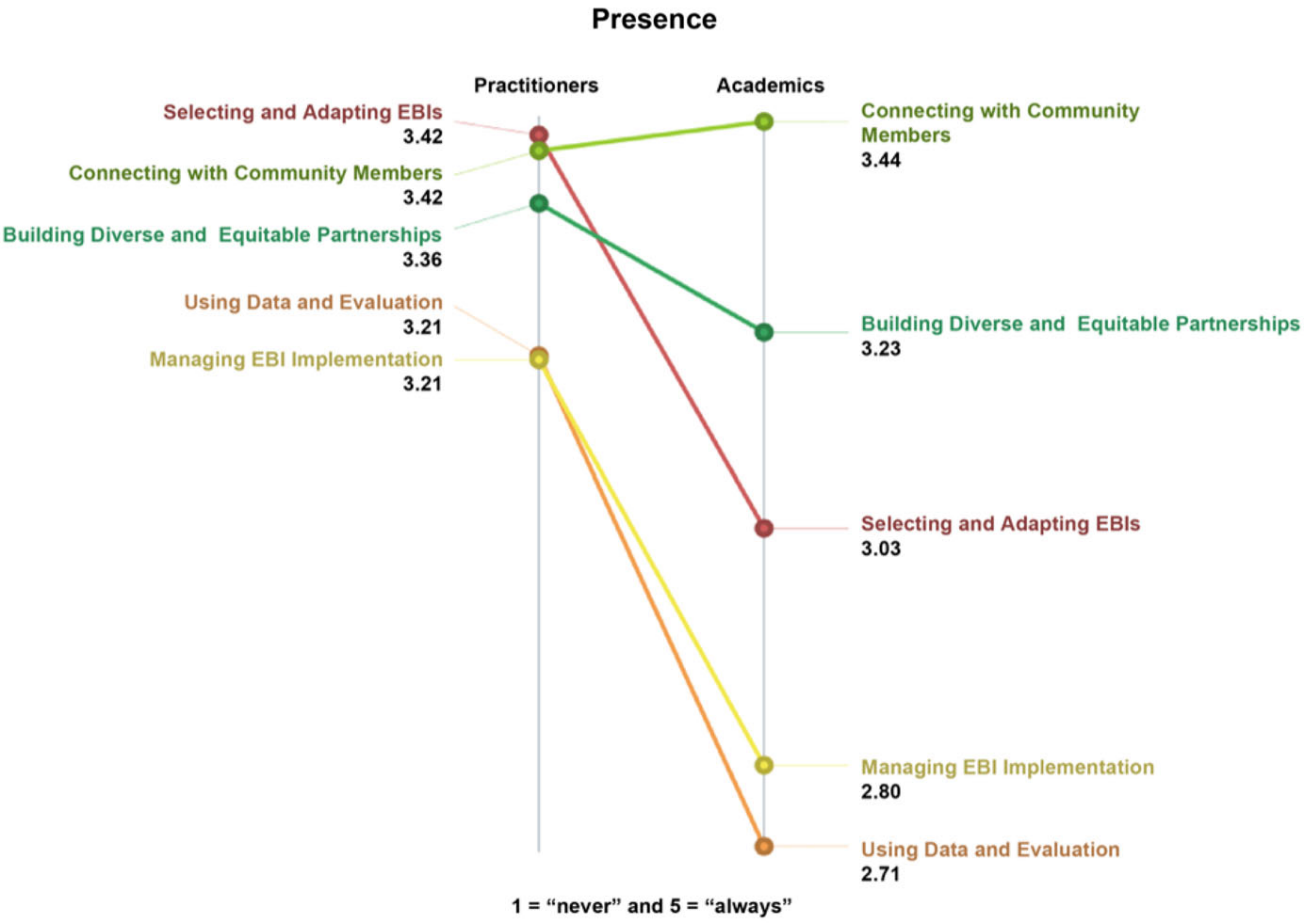
Perceived presence of skills for EBI use in CBOs, by skill cluster and participant type (*n* = 64 participants, 98 statements).

A similar examination of ratings for the presence of skills is presented in Fig. 5. Once more, CBO practitioners on average rated the presence of skills higher (overall *M* = 3.32, *SD* = 0.87, Range = 1.53) and within a narrower range than academics (overall *M* = 3.05, *SD* = 0.87, Range = 2.37). Although the level and range of the ratings were observed to be different, the distributions of average ratings were not. This suggests that CBO practitioners perceive a fairly similar level of presence for all five skills, compared to a broader gap for academics, e.g., between skills for connecting with community members compared to using data and evaluation. The ordering of the clusters (in terms of presence) was markedly different between the two groups. However, the pattern match correlation, which describes the pattern of average cluster ratings across groups, was 0.83, indicating a high magnitude of agreement between groups.

## Discussion

This study examined how academics and practitioners conceptualize the core skills CBO staff need to implement cervical cancer EBIs, with a focus on health equity. The model of core skills echoed many of the skills common to general models of EBI use but grouped them in different ways and also emphasized differences in skills for community member engagement versus partnership development and sustainment. The data also highlighted potential opportunities to support capacity-building through an extended focus on health equity in EBI use. Practitioner capacity to use EBIs is an important driver of implementation outcomes and, ultimately, health impact and is thus a critical area of focus [7,29,30].

We identified five distinct clusters based on how participants sorted the skills. Overall, the skills emphasized by participants were consistent with common models of EBI use: engaging stakeholders, using data and evaluation, adapting EBIs, and selecting an EBI [4,7]. In this study, participants separated connecting with community members from the broader range of partners involved with EBIs. This emphasis may reflect the inclusion of practitioners in this exercise. The formative work for this project and our early explorations of the data suggest that the five-cluster solution identified based on all participants’ data may mask important differences between academics and practitioners in their conceptualization of core skills and areas of need for capacity-building [11]. Similar to other group concept mapping studies involving academic and community groups [31], important distinctions in the conceptual thinking of the two subgroups may have implications for design, uptake, and sustainability of EBIs at the local level. Thus, the next set of analyses will explore this further and additional research to examine potential differences in definitions or understandings of skills is warranted.

Interestingly, we found statistically significant differences between the ratings of skills in each cluster in importance (higher) compared to presence (lower). This suggests a strong need for capacity-building interventions so that the presence of these skills in CBOs can match the level of importance. Both groups ranked “Managing EBI implementation” the lowest or second-lowest in terms of presence. One explanation may be the range of complex skills in this grouping, from finding funding for EBIs to understanding the mechanisms by which EBIs create behavior change. This complexity is echoed by models describing the multi-level determinants of implementation, from EBI characteristics to contextual barriers [32].

There are several skills in the high-importance, high presence quadrant, which suggests a strong foundation for skill-building. The roughly 15% of skills in the high-importance, low-presence quadrant are prime areas to focus on in capacity-building work. One-third of these skills were related to integrating equity considerations into EBI utilization. This reflects a broader trend in the field of implementation science to address equity considerations more explicitly and in a manner integrated into EBI utilization and research [33]. We also note that the 55 skills ranked as high importance could offer a useful set of targets for capacity-building given the ongoing training needs in CBOs due to changes in programming and turnover [13,34]. These skills are worth deeper examination in contrast to existing training curricula to identify areas of overlap and divergence [4]. The skills marked as low importance and presence offer insight into content that may not need to be addressed in capacity-building interventions, given the limited time CBO staff have for such activities.

Finally, we found that CBO practitioners’ ratings for the presence of skills had a smaller range across clusters than the academics’ ratings. This is worth further investigation as it may be an important source of disconnect if CBO practitioners perceive the core skills to be available at about the same level, but academics perceive a subset of skills as lacking in practice settings. Similarly, if the source of disconnect reflects distance of the practitioner or academic from actual implementation efforts, that will be important to understand. At the same time, the general overlap in rating order offers a useful point of agreement to build on. This point is worth exploring further, given that the participants all focused on cancer inequities.

As with any study, the findings must be placed in the context of strengths and limitations. One limitation of the study is that we used our networks and public listings to identify participants. Thus, we would not have been able to reach CBOs that are less visible and under-resourced. On a related note, the bulk of participants were from the Southern or Eastern US and there may be geographic variations that are unexplored in this study. However, the diversity of populations served by the CBOs in the sample (across a range of characteristics) suggests that we successfully recruited a diverse sample. The rigor of the analysis supports transferability to other settings in the US. We suggest this geographic boundary given the unique landscape for CBO activity in the USA, that may or may not be relevant in other countries. Another limitation is that important distinctions associated with each group might be difficult to see with the aggregation of multiple perspectives. For this reason, we will conduct subgroup analyses in the project’s next phase. Finally, the conceptual model presented here is bounded by the content generation process. We used standard models of EBI use as a starting point for our examination, and although many ideas were generated – presumably leading to some level of content saturation – it is still limited by those providing the input. At the same time, the study has several strengths. We were able to apply a group-based process to generate a constellation of ideas that are represented in the conceptual map presented here. By taking advantage of the positioning and relationships between nominated ideas, we could fully exploit the data set [23]. We recruited almost equal numbers of practitioners and academics (with a larger number of practitioners) and equally privileged their expertise. We also recruited a sufficiently large number of participants from each group, consistent with the recommendations found in the group concept mapping literature [28]. This study identified required skills that both groups of professionals agreed upon as important, as well as some meaningful differences between the importance and presence of clusters related to these skills. Another strength of this study was the focus on cancer equity. Most participants worked with marginalized groups, including racial/ethnic minorities, those identifying as LGBTQ+, as well as rural and low-income communities. These professionals were intentionally selected to develop a list of necessary skills tailored towards capacity-building in marginalized communities while maintaining cultural competence and feasibility within this work. Notably, the racial and ethnic diversity of the practitioner group was greater than the academic group. The broader range of positionalities of participants likely increased the diversity of contributions to the group concept mapping processes. Finally, the study used participatory processes for the group concept mapping method and the reliance on the expertise of practice-based advisors. This increases the likelihood that findings will be relevant and practical for practice settings [35]. Given that CBO practitioners have few professional development opportunities to build EBI skills [2], the importance of matching conceptual models becomes all the more important.

In conclusion, the findings from this study highlight the need to look beyond the traditional skills of evidence-based public health and evidence-based programming and identify ways to incorporate principles of health equity and community engagement to align goals. The work also points to a need to understand in greater detail how differences between practitioners and academics may be masked by a common mental model and where the greatest needs for capacity-building lie. In this way, capacity-building efforts will be more effectively aligned with equity-focused CBOs, setting the stage for greater impact of EBIs in communities that can benefit greatly from them.

## Supplementary material

For supplementary material accompanying this paper visit https://doi.org/10.1017/cts.2023.586. click here to view supplementary material
