# Supplementary material for: Priority skills for equity-focused, evidence-based cancer control in community-based organizations: A group concept mapping analysis with academics and practitioners
Source: J Clin Transl Sci. 2023 Jul 10;7(1):e164. doi: 10.1017/cts.2023.586 (PMC10425867; doi:10.1017/cts.2023.586)
Supplement: Supplementary file 1 [file S2059866123005861sup001.docx]

**Supplementary File 1. List of skills, sorted by cluster (n=98 skills, 5 clusters)**

| **Cluster** | **Skill number** | **Description** |
| --- | --- | --- |
| **1. Using data and evaluation** | 7 | how to effectively track adaptations |
|  | 10 | how to justify components of the intervention that are being adapted |
|  | 12 | know the steps to maintain fidelity when adapting the evidence-based intervention to suit the needs of the population |
|  | 28 | how to evaluate costs and benefits of the evidence-based intervention to inform return on investment and other economic evaluations |
|  | 40 | how to assess implementation context to inform selection of evidence-based intervention and implementation strategy |
|  | 47 | how to evaluate the implementation process with an equity lens |
|  | 48 | how to use evaluation to ensure equity in program reach |
|  | 49 | how to evaluate the process and impact outcomes for the evidence-based intervention |
|  | 50 | how to assess the implementation context to inform evaluation planning |
|  | 51 | how to build the evaluation from the beginning to continuously improve the work |
|  | 52 | how to develop and use ongoing monitoring, audit and feedback systems to collect data throughout the implementation |
|  | 53 | how to evaluate the adaptation of the intervention |
|  | 54 | how to gather, interpret and relate data findings in a clear and concise manner |
|  | 55 | how to obtain baseline rates and follow-up rates for the behavior in question |
|  | 57 | know to ensure quality and reliable, accurate data |
|  | 58 | know to monitor implementation quality and make improvements in implementation based on monitoring data |
|  | 59 | know what, when, and how to evaluate whether or not the evidence-based intervention is effective |
|  | 60 | understand the importance of collecting quantitative and qualitative information |
|  | 71 | how to assess need through conducting qualitative research like focus groups and interviewing |
|  | 72 | how to collect secondary local-level & state data |
|  | 73 | how to gather baseline data re: cervical cancer screening needs |
|  | 76 | how to measure client knowledge, awareness, and attitudes towards cervical cancer as part of the initial environmental assessment |
| **2. Selecting and adapting EBIs** | 1 | how to adapt evidence-based interventions for underserved populations to ensure they are culturally responsive |
|  | 3 | how to adapt evidence-based interventions to local culture (language, values) |
|  | 4 | how to adapt evidence-based interventions to meet the needs/capacity of the CBO |
|  | 5 | how to adapt the evidence-based intervention materials to ensure a regular person is going to be able to understand it |
|  | 6 | how to adapt the intervention to their specific population/situation without losing the fidelity of the intervention |
|  | 8 | how to identify what could be adapted to better tailor services to the population of focus |
|  | 9 | how to iteratively adapt the evidence-based intervention throughout its implementation to address the emerging needs and interests of the population |
|  | 14 | how to identify community barriers to accessing care or participating in evidence-based interventions |
|  | 15 | how to identify solutions to address community barriers to access to evidence-based interventions |
|  | 16 | how to identify barriers to implementing the evidence-based intervention within their selected population |
|  | 33 | how to ensure that all aspects of the evidence-based intervention are culturally appropriate: from communication type to properly translated assessments and measures |
|  | 42 | how to effectively choose an evidence-based intervention that is particular to their target population (e.g., rural, urban, race, age, LGBTQ, etc.) |
|  | 74 | how to identify the assets and needs of the communities served by the CBO |
|  | 75 | how to incorporate the social determinants (race, income, employment, insurance) into needs assessment for populations |
|  | 79 | know what has worked/hasn't worked in the community in the past |
| **3. Connecting with community members** | 11 | how to support authentic participation of the community in the adaptation process |
|  | 21 | understand the historical, lived, and current experiences of this community and communities like this one |
|  | 22 | how to center the community that they seek to impact/ serve |
|  | 23 | how to engage at-risk populations with different cultures and languages |
|  | 24 | know how to cultivate and maintain trust with the community |
|  | 25 | know to ensure that the community is not just participating in the evidence-based intervention but also fully informed and educated as to what its purpose, intent, and content is |
|  | 26 | know to listen to the community you are trying to serve and be prepared to make changes |
|  | 31 | how to embed cultural competency into their work and interactions with communities |
|  | 32 | how to engage priority populations in a culturally responsive way |
|  | 45 | how to create bi-directional channels of communication with communities being served to get insight, feedback, and buy-in |
|  | 46 | how to incorporate diverse perspectives, particularly of residents' voices, throughout the evidence-based intervention process |
|  | 61 | how to ensure the community gives input on the language/terms used |
|  | 62 | how to communicate with diverse participants in a way that is culturally and linguistically appropriate |
|  | 63 | how to engage priority population in a linguistically responsive way |
|  | 65 | how to use inclusive messaging, e.g., people with a cervix |
|  | 66 | how to use plain language to provide health education to the community |
|  | 70 | how to relate to people with empathy and compassion |
| **4. Building diverse and equitable partnerships** | 2 | how to access people who can help with material adaptations (graphics, design, translation, accessibility, etc.) to ensure cultural relevance |
|  | 13 | how to engage in advocacy and coalition-building work |
|  | 17 | how to get buy-in from the local community |
|  | 18 | how to make the "case" to their leadership, administration, and other staff to embark on a program to implement evidence-based interventions |
|  | 19 | know to build champions across all supporters to get institutional buy-in |
|  | 20 | know to make sure there is buy-in with senior leadership and staff that are part of the implementation |
|  | 37 | know where to find assistance if needed, whether it is a peer organization, an academic partner, someone from within one's organization, or a partner with knowledge of evidence-based programming |
|  | 64 | how to share information through social media and community events |
|  | 67 | understand basic background ideas of public/community health |
|  | 80 | how to lead implementation efforts through team-building, organizational leadership, and ongoing support |
|  | 81 | how to create community-clinical linkages with local/regional health systems |
|  | 82 | how to partner with organizations who work closely with the community or represent people with lived experience |
|  | 83 | how to approach collaborating with their local academic institution to utilize the public health and evaluation expertise if they do not internally have those skills/staff |
|  | 84 | how to build relationships with health care providers who will provide services |
|  | 85 | how to demonstrate dependability & trustworthiness to partners |
|  | 86 | how to develop formalized processes with partners through MOUs/BAAs, workflows, and best practices |
|  | 87 | how to develop partnerships with and across diverse stakeholder groups |
|  | 88 | how to engage stakeholders throughout the intervention to retain their investment |
|  | 89 | how to equitably structure stakeholder partnerships (power, trust, transparency, ongoing commitment, resource sharing, etc.) |
|  | 90 | how to strategically link with partners that can address capacity and resource gaps |
|  | 91 | know to promote a sense of ownership and commitment in stakeholders through power sharing and sustained engagement throughout the process |
|  | 92 | how to use implementation efforts to rebalance power |
|  | 94 | how to understand organizational and community infrastructure and resources |
| **5. Managing EBI implementation** | 27 | how to be realistic about budget and how much it will cost to implement a cervical cancer program |
|  | 29 | how to find resources and funding for the staff and infrastructure necessary to implement the intervention |
|  | 30 | how to obtain funding to implement the evidence-based intervention |
|  | 34 | How to report and disseminate/communicate evaluation findings to different stakeholders in appropriate formats |
|  | 35 | know enough about the intervention |
|  | 36 | know what evidence-based interventions look like in practice |
|  | 38 | know why certain interventions are evidence-based and why some are not |
|  | 39 | how to systematically deliver the proposed intervention |
|  | 41 | how to determine if the evidence-based intervention will be able to be successfully implemented in their organization |
|  | 43 | understand the logic and mechanisms, implementation, and outcomes of candidate evidence-based interventions |
|  | 44 | understand the source and strength of the evidence for the intervention |
|  | 56 | know about quality improvement principles, PDSAs - small test of change |
|  | 68 | how to address complex questions about cervical cancer or know where to look for additional information if you do not have the answer |
|  | 69 | how to promote behavior change at the individual, interpersonal, organizational, and systems levels |
|  | 77 | how to use systems mapping tools |
|  | 78 | how to learn from other groups that have successfully used this type of evidence-based intervention |
|  | 93 | how to develop strategies for staff turnover since that can have bearing on the fidelity of the intervention and therefore effectiveness |
|  | 95 | how to plan for sustainability from the beginning |
|  | 96 | know if the evidence-based intervention is sustainable, e.g., there are enough resources to sustain it |
|  | 97 | how to access appropriate technical assistance and implementation guidance as needed |
|  | 98 | how to establish a work plan to keep track of all activities related to the evidence-based intervention: who is responsible for what, what defines success, deadlines, etc. |
